# NK Cell Metabolism and TGFβ – Implications for Immunotherapy

**DOI:** 10.3389/fimmu.2019.02915

**Published:** 2019-12-13

**Authors:** Karen Slattery, Clair M. Gardiner

**Affiliations:** School of Biochemistry and Immunology, Trinity Biomedical Sciences Institute, Trinity College, Dublin, Ireland

**Keywords:** NK cells, metabolism, mitochondria, TGFβ, immunotherapy

## Abstract

NK cells are innate lymphocytes which play an essential role in protection against cancer and viral infection. Their functions are dictated by many factors including the receptors they express, cytokines they respond to and changes in the external environment. These cell processes are regulated within NK cells at many levels including genetic, epigenetic and expression (RNA and protein) levels. The last decade has revealed cellular metabolism as another level of immune regulation. Specific immune cells adopt metabolic configurations that support their functions, and this is a dynamic process with cells undergoing metabolic reprogramming during the course of an immune response. Upon activation with pro-inflammatory cytokines, NK cells upregulate both glycolysis and oxphos metabolic pathways and this supports their anti-cancer functions. Perturbation of these pathways inhibits NK cell effector functions. Anti-inflammatory cytokines such as TGFβ can inhibit metabolic changes and reduce functional outputs. Although a lot remains to be learned, our knowledge of potential molecular mechanisms involved is growing quickly. This review will discuss our current knowledge on the role of TGFβ in regulating NK cell metabolism and will draw on a wider knowledge base regarding TGFβ regulation of cellular metabolic pathways, in order to highlight potential ways in which TGFβ might be targeted to contribute to the exciting progress that is being made in terms of adoptive NK cell therapies for cancer.

## The Importance of Immunometabolism

Over the past decade, the field of immunometabolism has exploded and become one of the fastest growing research areas in immunology. We now appreciate the vast impact that metabolism has on the fate and function of immune cells, although we are far from fully understanding it. The metabolic status of a cell dictates the functions that it can carry out e.g., increased mitochondrial mass and high rates of fatty acid oxidation are fundamental for memory T cells to carry out their antigen recall response (1). Immunometabolism has reinvigorated many areas of research and allowed older concepts to be viewed in a new light.

Importantly, immunometabolism is having an impact in the clinic. The effect that old drugs have on the metabolism of immune cells is now being explored for the first time. For example, metformin, which has been used to treat type 2 diabetes for many decades, works by inhibiting complex I in the electron transport chain and hence can strongly impact immune cells that use oxidative metabolism to carry out their functions (2). Indeed, recent research has shown that metformin can skew T cell differentiation toward regulatory and memory T cells (3). Another example is the deeper understanding provided by metabolism of how checkpoint inhibitors work during cancer therapy. Bengsch et al. demonstrated that anti-PDL1 therapy reprogrammed metabolism of exhausted T cells and improved several readouts of mitochondrial structure and function as part of the mechanisms of action of this therapy (4). It is likely that these checkpoint inhibitors are also impacting the metabolism of Natural Killer (NK) cells, as NK cells also express some of these checkpoint antigens (5).

Many of the new concepts in immunometabolism have been inspired by the more advanced field of cancer metabolism. For nearly a century we have been aware of the metabolic reprogramming that takes place in tumor cells (6), and this has resulted in the development of drugs that target tumor cell metabolism e.g., methotrexate, an inhibitor of dihydrofolate reductase, that has been used in chemotherapy since the 1940s (7). These drugs were designed based almost exclusively on research on tumor cell metabolism and their potential impact on immune cells was not investigated or considered. However, as we increasingly turn to the immune system for new cancer therapy approaches, a trend likely to continue given the high rewards yielded to date, cancer researchers and immunologists need to work together to consider the consequences of strategies proposed for synergy to be achieved. For example, rapamycin [a mammalian target of rapamycin complex 1 (mTORC1) inhibitor], is currently in clinical trials for neuroblastoma (ClinicalTrials.gov; NCT01331135) and for breast cancer (ClinicalTrials.gov; NCT02536625). We predict that this therapy will severely inhibit NK cell and other cytotoxic immune cell responses, which are highly dependent on mTORC1 mediate metabolic reprogramming to carry out their functions. This is particularly concerning for patients where antibody mediated therapy is part of their standard treatment of care e.g., anti-GD2 therapy for high risk neuroblastoma and trastuzumab for HER2^+^ breast cancer, and which works in part by promoting NK cell antibody dependent cellular cytotoxicity (ADCC) (8). We are only now beginning to understand the off-target effects that these metabolic cancer therapies have on the immune system and a current goal of the field is trying to figure out ways in which we can selectively target cancer metabolism while protecting the anti-tumor immune response.

What is more exciting, however, is that immunometabolism research is revealing new drug targets that may prove effective in the treatment of a variety of human diseases. As we now know that metabolic inhibitors such as 2-deoxyglucose (2DG) can inhibit the immune response, the potential for these to be used in preventing graft rejection is under investigation, and pre-clinical results from mouse models are very promising (9). Sukumar et al. showed that inhibiting glycolytic metabolism in CD8^+^ T cells increases the generation of memory cells and their anti-tumor functions (10), while Fisicaro et al. showed that improving mitochondrial fitness using antioxidants is sufficient to revive exhausted CD8^+^ T cells and increase their antiviral functions (11). In human sepsis patients, IL7 treatment increases glucose metabolism and mTOR activity in dysfunctional T cells (12). Overall, it seems likely that some of these immunometabolism-derived therapies will reach clinical trials in the near future.

## Specific Immune Cells Adopt Particular Metabolic Configurations

**Figure 1:**
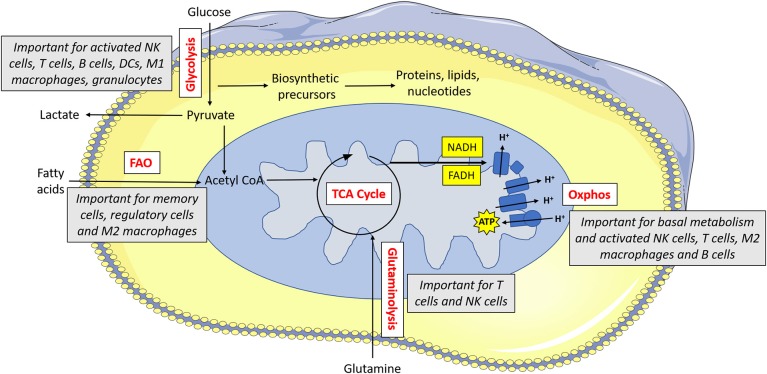
Metabolism drives immune cell function. Glucose is metabolized by glycolysis, which is essential for activated NK cells, T cells, B cells, dendritic cells (DCs), M1 macrophages and granulocytes. Pyruvate can be converted to lactate and secreted from the cell or else it can be converted to acetyl CoA which feeds into the TCA cycle. The TCA cycle results in the production of reducing equivalents (NADH, FADH) which feed into the electron transport chain. The electron transport chain uses the electrons supplied by NADH and FADH to pump protons across the inner membrane. This force is then used to drive ATP synthase which makes ATP. Oxphos is important for immune cells when they are at rest, and it is also essential for activated NK cells, T cells, M2 macrophages and B cells. Acetyl CoA can alternatively be supplied by fatty acids—this form of metabolism is important for memory cells, regulatory cells and M2 macrophages. Glutamine can feed into the TCA cycle via glutaminolysis—this pathway is used by T cells and to a lesser extent, NK cells.

Indeed, there is a growing appreciation of the extent to which immunometabolism underpins many aspects of the immune response. Different immune cells have specialized metabolic configurations that allow them to carry out their specialized functions. In general, these pathways provide both energy (in the form of adenosine tri-phosphate; ATP) and biosynthetic precursor molecules that will be required for carrying out their effector functions. Various fuels can be used by cells including glucose, glutamine and fatty acids. As mentioned, memory T cells mainly engage in fatty acid oxidation. Fatty acid oxidation involves the breakdown of cellular fatty acids into smaller components which can then go into the mitochondria of the cell where they feed the tricarboxylic acid (TCA) cycle to facilitate NADH and FADH_2_ production (see Figure 1 for main cell energy production pathways) which are required to supply electrons to the mitochondria. This final process called oxidative phosphorylation (or oxphos) consumes oxygen and results in production of ATP. Unlike glucose, fatty acids can easily be stored as lipid droplets within the cell. As such, memory T cells synthesize fatty acids so that they can rapidly oxidize the stored fatty acids which then supplies the energy required for the recall response (13). Similarly, regulatory T cells use fatty acid oxidation to support their long life span (14). Other immune cells, with different functions, use alternative forms of metabolism. At rest, most naïve immune cells carry out oxphos to meet their homeostatic needs. Once an immune cell becomes activated, it reprograms cellular metabolism to suit it functions. In many cell types, this involves upregulation of glycolysis, the key pathway in glucose metabolism. This increased glycolytic flux supports rapid ATP synthesis in addition to production of various biosynthetic precursors such as amino acids (15). As this upregulation of glycolysis takes place in the presence of oxygen, it is known as aerobic glycolysis, and is fundamental for the functions of NK cells, effector T cells, B cells, dendritic cells (DC), neutrophils, and M1 macrophages (16). By understanding how these metabolic pathways support functions of the immune response, we can aspire to unlock new ways in which to control and regulate the immune system therapeutically.

## NK Cell Metabolism

NK cells have characteristic metabolic configurations which facilitate their functions. Oxphos is used to meet their homeostatic needs, and after short term stimulation (6 h) oxphos remains the dominant form of metabolism (17). However, when activated over longer periods of time (18h+), NK cells upregulate both oxphos and glycolysis, with their main form of metabolism shifting to glycolysis (18, 19). This shift allows for production of biosynthetic precursors that are needed for NK cells to carry out their functions and may also serve to enhance their longevity and thus their ability to function in parallel with the adaptive immune system.

**Figure 2:**
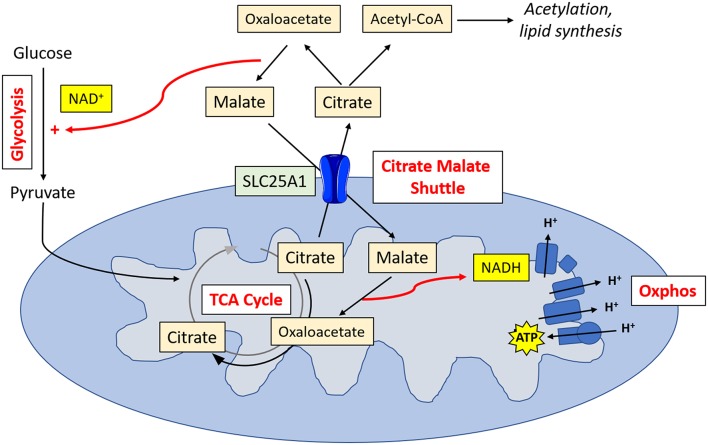
NK cell metabolism. Activated NK cells metabolize glucose to pyruvate. Pyruvate is converted to acetyl CoA which is then converted into citrate. Citrate is exported into the cytosol via SLC25A1, where it is converted into oxaloacetate and acetyl CoA. Acetyl CoA can then be used in acetylation reactions or for lipid synthesis. Oxaloacetate is converted back in malate, resulting in the production of NAD^+^, and essential cofactor for glycolysis. Malate is transported back into the mitochondria, where it is converted back into oxaloacetate, producing NADH which can then feed into the electron transport chain for ATP synthesis.

Metabolic tracing experiments have revealed that NK cells adopt a unique metabolic configuration, whereby they use the citrate-malate shuttle to fuel oxphos (Figure 2). Instead of cycling citrate through the TCA cycle like most cells, they export it to the cytosol where it is then converted to acetyl CoA and oxaloacetate (20). The acetyl CoA can then be used for acetylation reactions (important for innate immune training) or to produce fatty acids, while the oxaloacetate is converted back to malate and then shuttled back to the TCA cycle. Ultimately, this results in the production of cytosolic NAD^+^, which is an essential cofactor for the glycolytic enzyme glyceraldehyde 3-phosphate dehydrogenase (GAPDH), and NADH, which is fed into the electron transport chain support ATP production. This facilitates increases in both glycolysis and oxphos which are required for the NK cell functional response.

At the mitochondrial level, cytokine stimulation can also induce significant changes. Miranda et al. looked at IL2 induced mitochondrial changes in human NK cells (21). They showed that IL2 stimulation increased both the mitochondrial mass and the mitochondrial membrane potential. This was dependent on the expression of peroxisome proliferator-activated receptor gamma coactivator 1-alpha (PGC-1α), and its deletion resulted in reduced mitochondrial mass and membrane polarization, in addition to impaired IFNγ production. Abarca-Rojano et al. showed that the mitochondria of NK cells depolarize upon contact with a tumor cell, and that they reorganize themselves to the point of contact with the cancer cell (22). It has also been shown that mitophagy, i.e., the removal of defective mitochondria, is essential for the development of NK cell memory (23). Interestingly, this process occurs in a reactive oxygen species (ROS) dependent manner.

## Regulation of NK Cell Metabolism

The complex metabolic configuration of NK cells requires a high degree of regulation. As with many immune cells, several aspects of NK cell metabolism are highly dependent on mTORC1, a protein complex that functions as a nutrient/metabolic sensor and coordinates protein synthesis. mTORC1 activity increases upon cytokine stimulation of NK cells and enhances glycolytic flux through the cells. Inhibition of mTORC1 using rapamycin results in inhibition of cell size, nutrient receptor expression (CD71 and CD98), and glycolysis, but not oxphos (18, 19, 24). mTORC2 is closely related to mTORC1 and they share the mTOR kinase subunit; however, mTORC2 associates with different subunits, has different downstream targets and is insensitive to acute inhibition by rapamycin. Phosphorylation of mTOR (mTORC1 and/or mTORC2), has been shown to correlate with NK cell maturation in the bone marrow and the spleen (24). Furthermore, Yang et al. showed that mTORC1 and mTORC2 both play a role in regulating murine NK cell maturation using Raptor (subunit of mTORC1) and Rictor (subunit of mTORC2) conditional knock out mice (25). Interestingly, while mTORC1 promoted mTORC2 activity, mTORC2 repressed mTORC1 activity, thus inhibiting NK cell functions and SLC7A5 (amino acid transporter) expression (26).

Beyond mTOR signaling, NK cells have quite unique underlying regulatory mechanisms. Sterol regulatory element-binding protein (SREBP) is a transcription factor known traditionally for its role in fatty acid and cholesterol synthesis. However, in NK cells, SREBP is fundamental for the modulation of glycolysis and oxphos. This is partly due to regulated expression of the main transporter involved in the citrate-malate shuttle—SLC25A1 (20). Unsurprisingly, inhibition of SREBP also inhibits NK cell functions.

cMyc is another transcription factor that has been shown to be essential for NK cell metabolism (27). In this setting, cMyc is acutely controlled by the availability of amino acids. These amino acids, glutamine in particular, are imported into the cell via the transporter SLC7A5 where they then turn on cMyc and allow it to regulate NK cell metabolism and function. Interestingly, neither HIF1α nor glutaminolysis were shown to be important for NK cell metabolism in this study, which is in contrast to other cytotoxic lymphocytes. Dong et al. has recently shown that inositol-requiring enzyme 1 (IRE1), an ER-nucleus signaling protein, is essential for optimal NK cell anti-tumor and anti-viral responses and works by directly regulating cMyc activity (28).

## TGFβ

As proinflammatory cytokines such as IL2, IL12, and IL15 induce robust NK cell metabolic responses like those described above, anti-inflammatory cytokines can also inhibit them. For decades, many studies have reported on the negative effects that TGFβ has on NK cell functions (29–31). TGFβ is a pleiotropic cytokine and can play a role in a wide variety of processes, including differentiation, migration, apoptosis, wound healing and angiogenesis (32). While TGFβ is generally acknowledged as a negative regulator of growth (e.g., via inhibition of cMYC and CDKs) (33), it can also promote proliferation of certain cells (e.g., mesenchymal stem cells) (34).

The TGFβ canonical signaling pathway is relatively simple—TGFβ first binds to TGFβ receptor 2 (TGFβR2), which leads to the recruitment, transphosphorylation and activation of TGFβ receptor 1 (TGFβR1). TGFβR1 then phosphorylates its downstream targets using its cytoplasmic kinase domain to initiate the SMAD dependent signaling pathway [5]. Phosphorylated SMAD2 and SMAD3 bind to SMAD4 and the complex then travels to the nucleus where it modulates gene expression and directs the cells response to TGFβ. SMAD6 and SMAD7 are negative regulators of this pathway, and their expression may be induced by SMAD4-2/3 forming a negative feedback loop (35). Ubiquitination of TGFβ signaling molecules is a common way in which this pathway is regulated (36).

Although there are relatively few SMAD proteins involved in TGFβ signaling, they commonly regulate gene expression in cooperation with hundreds of high affinity DNA-binding transcription factors and transcription coregulators. These are controlled by and often dependent on other signaling pathways, resulting in highly context-dependent transcriptional responses being controlled by signaling cross talk (37, 38).

Alternatively, TGFβ ligands can signal through non-SMAD (“non-canonical”) signaling pathways. This entails recruitment and activation of signaling mediators by ligand-occupied receptors i.e., TGFβR1 and TGFβR2 (39). These pathways include branches of PI3K-Akt signaling which activate the MAPK pathway and mTOR, small GTPase pathway, JNK/p38 pathway, and can be regulated by phosphatases such as PP2A (39–41). Many of the signaling molecules in these pathways interact directly with TGFβR1 and/or TGFβR2 e.g., TRAF6 (42, 43). Of note, while the impact of TGFβ canonical signaling on NK cells is well described, the effect of TGFβ non-canonical signaling on NK cells remains largely unexplored.

## TGFβ and NK Cell Metabolism

While it has been known for many decades that TGFβ inhibits the activity and functions of NK cells (30, 44, 45), the molecular mechanisms underlying this have remained poorly understood. Scientists are beginning to investigate these and immunometabolism studies may help shed some light on this topic. Viel et al. showed that while TGFβ had no effect on the development of murine NK cells, genetic deletion of the TGFβR2 (specifically in NK cells via Ncr1^Cre^ mice crossed with TGFβR2^fl/fl^ mice) reduced tumor metastasis in two tumor models and increased nutrient receptor expression and mTORC1 activity in IL15 stimulated NK cells (46). *In vitro*, TGFβ treatment inhibited mouse and human metabolic responses (oxphos and glycolysis), nutrient receptor expression and mTORC1 activity. Rapamycin treatment recapitulated most of the effects of TGFβ treatment. In many cases, the effects of TGFβ were more potent than those of rapamycin, yet mTORC1 deletion had more significant effects than constitutive TGFβ signaling, suggesting that mTORC1 is not the only pathway involved in the repression of NK cell activity by TGFβ.

Work from our lab also investigated the effect of TGFβ on human NK cells stimulated with cytokine (47). We showed that NK cells stimulated with IL2 for 18 h in the presence of TGFβ had significantly reduced levels of oxphos, maximal respiration and glycolytic capacity. Interestingly, glycolysis was not affected. Furthermore, the expression of CD69, CD71 and the functional mediators IFNγ and granzyme B were also reduced following TGFβ treatment. With the exception of granzyme B, these effects were reversed by adding a TGFβR1 inhibitor, suggesting that TGFβ's impact on granzyme B is mediated by an alternative pathway, likely one of the non-canonical pathways that signals through TGFβR2 only. Indeed, deletion of the TGFβR2 (specifically in NK cells) has been shown to reduce granzyme B expression in murine NK cells (46).

In contrast to Viel et al. we observed that TGFβ had no impact on NK cell mTORC1 activity (at 30 min, 1 or 18 h). However, if the NK cells were exposed to TGFβ for a prolonged period of time (5 days), TGFβ reduced IL2 induced mTORC1 activity. There are several potential explanations for the discrepancies between these two studies. First and foremost, the vast majority of the Viel et al. study was carried out on murine NK cells. In that study, the impact of TGFβ on human NK cell mTORC1 activity was evident in only some donors and was modest compared to its effect on murine NK cell mTORC1 activity. Finally, the timepoints (mainly 1 vs. 18 h) and cytokine stimulations (IL15 vs. IL12/15) differed between the studies, precluding direct comparisons.

In contrast to both above studies, there are many reports showing that TGFβ actually activates mTORC1 (and mTORC2) in non-immune cells. Rahimi et al. showed that TGFβ activates mTORC1 mediated growth in fibroblasts (but not epithelial cells), and that mTORC2 is required for TGFβ mediated Akt signaling (48). More recently it was shown that TGFβ promotes mTORC1 mediated GLUT1 expression and glycine biosynthesis in human lung fibroblasts, which culminates in collagen production and fibrosis (49). Similarly, others have reported that TGFβ induces mTORC1 activity and subsequent HIF1α activity and collagen expression in human kidney cells (50, 51). Cheng et al. showed that TGFβ induced epithelial-mesenchymal transition cell proliferation and migration is dependent on mTORC1 in cervical carcinoma cells (52). Indeed, it seems there is still a lot to be learned about the relationship between TGFβ and mTORC1 (and mTORC2) signaling. It will be interesting to see in the future whether TGFβ affects the mTORC1 activity of different immune cells in distinct ways.

Beyond the interplay of TGFβ and mTORC1, another study has demonstrated the impact of TGFβ on the expression of the anabolic enzyme fructose-1,6-bisphosphatase (FBP1) in murine NK cells (53). FBP1 is an essential enzyme in gluconeogenesis, a pathway which results in the generation of glucose from non-carbohydrate substrates such as amino acids, triglycerides and lactate. FBP1 hydrolyses fructose 1,6-bisphosphate into fructose 6-phosphate, thus reversing the direction of the glycolysis pathway and promoting glucose synthesis. While gluconeogenesis is traditionally known to take place in the liver and serve to maintain blood glucose levels, this study showed that TGFβ treatment (24 h) increased the expression of FBP1 mRNA in murine lung NK cells, which induced NK cell dysfunction and tumor progression. Whether TGFβ has such effects on other glycolytic/metabolic enzymes in NK cells is yet to be investigated. This could potentially shed light on TGFβ induced metabolic defects described in the above studies.

Recent work from our lab showed that peripheral blood NK cells from patients with metastatic breast cancer have severely reduced levels of metabolism and impaired mTORC1 activity (54). Neutralization of TGFβ *ex vivo* restored levels of oxphos, mTORC1 activity, nutrient receptor expression and importantly, IFNγ production. TGFβ neutralization did not restore IL2 induced glycolysis—however, we previously reported that TGFβ treatment had no effect on glycolysis in human NK cells (47). Hence, TGFβ does not seem to impact oxphos and glycolysis in the same manner. The overnight restoration of various metabolic and functional parameters of NK cells from breast cancer patients gives promise to the various TGFβ targeted therapies currently in development.

## Potential Roles for TGFβ in Regulating NK Cell Metabolism

**Figure 3:**
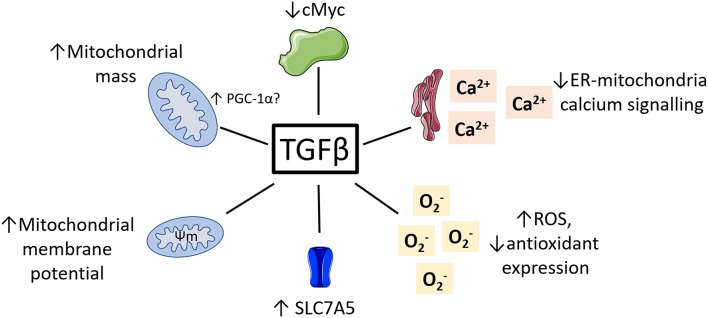
Potential roles for TGFβ in regulating NK cell metabolism. TGFβ has been shown to impact the metabolism of various non-immune cell types. This included reduced cMyc activity, reduced ER-mitochondrial signaling, increased ROS and reduced antioxidants, increased mitochondrial membrane potential and increased mitochondrial mass.

While research on the role of TGFβ regulating NK cell metabolism is in its infancy, there is a vast body of literature detailing the impact of TGFβ on metabolism in other cell types. Given the complexity of TGFβ signaling and its pleiotropic effects on many different cell types, these studies are unlikely to provide a simple understanding of what is happening in NK cells. However, they provide a strong starting point and illustrate several molecular mechanisms which may underlie TGFβ's negative impact on NK cell metabolism and function. Here, we consider some key examples of how TGFβ might be affecting NK cell cellular metabolism and suggest ways in which we might use this knowledge to improve immunotherapy (see Figure 3).

### TGFβ and cMyc

As described above, cMyc is an important regulator of NK cell function and metabolism (27). It has long been known that one of the main ways in which TGFβ acts a growth repressor is via inhibition cMyc (55). Indeed, TGFβ has been shown to inhibit cMyc expression via the canonical signaling pathway in several cell types including keratinocytes (56), tumor cell lines (57, 58) and oligodendrocyte progenitors (59). Hence, it is possible that TGFβ is affecting cMyc expression in NK cells and that this is contributing to the reduced metabolism and functions observed in (46) and (47). Interestingly, Zakiryanova et al. recently reported reduced cMyc expression in NK cells from human lung and gastric cancer patients. As we know that TGFβ levels are commonly increased in patients with these cancers (60–62), TGFβ-mediated cMyc inhibition may be an underlying cause for the NK cell dysfunction observed in these cancers (53, 63, 64). If this is the case, alleviating cMyc suppression may bypass some of the inhibitory effects that TGFβ is having on NK cell metabolism. For example, increasing the availability of amino acids will stabilize cMyc, or inhibition of glycogen synthase kinase 3 will reduce cMyc degradation. Both approaches have previously been shown to increase NK cell activity and function in mice and humans (27, 65, 66).

TGFβ itself has not been shown to directly regulate the activity of SREBP (another essential regulator of NK cell metabolism). However, there are several reports showing that SREBP regulates TGFβ activity in kidney cells (67–69). Thus, it is interesting to speculate that there may be a role for altered SREBP activity in NK cells leading to dysregulated TGFβ activity in cancer.

### TGFβ and ROS

ROS are highly reactive, oxygen containing molecules such as ${\text{O}}_{2}^{-}$, OH^−^ and H_2_O_2_. They are produced in the cytosol, e.g., as a by-product of NADPH oxidase enzymes, or in the mitochondria, e.g., by complex 1 and 3 of the electron transport chain. Cells express various antioxidant proteins such as superoxide dismutase (SOD) and catalase which can convert ROS into water and oxygen. ROS can be produced under homeostatic conditions and are involved in many biological processes such as redox signaling pathways and apoptosis. In murine NK cells, they play an essential role in the generation of NK cell anti-viral memory (23). However, excessive ROS can be very harmful for a cell as the ROS can react with and damage various cellular components such as DNA and protein. Excessive ROS may also result in aberrant cell signaling through oxidative modification of redox-sensitive signaling proteins e.g., MAPK, HIF or NFkB (70). Recent studies showed that excessive ROS production is associated with NK cell metabolic dysfunction in metastatic breast cancer patients and in childhood obesity (54, 71). Interestingly, there is significant evidence demonstrating a connection between TGFβ and ROS production, with TGFβ treatment stimulating ROS production in many different studies.

The ability of Treg cells to suppress proliferation of T effector cells was enhanced by TGFβ treatment, and this was reversed via the addition of the antioxidant n-acetylcysteine (NAC) or by an inhibitor of NADPH oxidase (72). In part, this may be mediated by the protein GARP, which is induced on activated Tregs and anchors latent TGFβ to the cell surface (73). In airway smooth muscle cells, TGFβ induced the expression of NADPH oxidase 4 (NOX4) thus leading to cytosolic ROS production (74). Michaeloudes et al. confirmed this result, and also showed that TGFβ reduces expression of the cytosolic antioxidant catalase and of the mitochondrial antioxidant SOD2 (75). In lung epithelial cells, TGFβ signaling through the TGFβR1 has been shown to drive mitochondrial ROS production. This coincided with decreased complex IV activity, and inhibition of complex IV recapitulated the effects of TGFβ in terms of ROS production (76). Similarly, Jain et al. showed that TGFβ treatment increased mitochondrial and cytosolic ROS in lung fibroblast cells (77). In this case, mitochondrial ROS was produced by complex III and stimulated the expression of NOX4, in addition to other TGFβ target genes. Finally, in bone marrow mesenchymal stem cells, TGFβ treatment increased production of mitochondrial ROS and decreased expression of SOD2 (78).

Taking these studies together, it seems likely that TGFβ might impact the antioxidant system of NK cells and that this may be altering their function and/or metabolism. If this proves to be true, modulating NK cells such that they are more resistant to oxidative stress (e.g., by pre-treating with antioxidants or overexpressing antioxidant enzymes such as SOD2) may help them to cope with TGFβ induced ROS. Hence, in a TGFβ rich environment such as the tumor microenvironment, modified NK cells will be more resistant to oxidative damage and more able to fight the tumor. Interestingly, pre-treatment of exhausted CD8^+^ T cells with antioxidants has been shown to restore their antiviral activity (11) during human chronic hepatitis B infection, where systemic increases in TGFβ have been reported (79, 80).

The interplay between TGFβ and ROS is of particular interest in the context of murine NK cell memory where we know ROS plays a major role (23). Here, ROS production is required for the removal of defective mitochondria and the development of NK cell anti-viral memory. Perhaps in this case, TGFβ might play a positive role by stimulating ROS production in faulty NK cells and promoting their clearance by mitophagy.

### TGFβ and Mitochondrial Structure and Function

It is widely acknowledged that TGFβ plays a key role in regulating mitochondrial apoptosis (81), and as described above, TGFβ can stimulate mitochondrial ROS production in a variety of cell types. However, there are many other ways in TGFβ can modulate mitochondrial activities. TGFβ was reported to increase mitochondrial mass in primary human lung fibroblasts (82). In this study, TGFβ signaling via the canonical pathway culminated in the upregulation of PGC-1α expression. Interestingly, PGC-1α mediated increases in mitochondrial mass and polarization has been shown to be essential for NK cell IFNγ production (21).

Mitochondrial membrane potential is the electronegative force across the inner mitochondrial membrane that drives ATP production using ATP synthase. Yoon et al. reported that TGFβ increased the mitochondrial membrane potential of lung epithelial cells, yet interestingly did not alter secretion of apoptotic factors from the mitochondria (76). Similarly, TGFβ has been shown to increase mitochondrial membrane potential in mouse kidney cells (83). Indeed, it is tempting to speculate that the increased mitochondrial mass and membrane potential observed in NK cells from metastatic breast cancer patients is in part due to increased TGFβ activity (54). In breast cancer cells, TGFβ has been shown to regulate expression of mitochondrial uncoupling protein 2 (UCP2), a mitochondrial transporter involved in dissipation of the mitochondria membrane potential to facilitate heat production (84).

As discussed, NK cells treated with TGFβ have reduced levels of oxphos and maximal respiration (47). This has also been shown in hepatocellular carcinoma cells, where TGFβ treatment skewed metabolism away from oxphos in order to promote the epithelial/mesenchymal cell transition (85). Interestingly, the expression of SLC7A5 was increased on the cancer cells in response to TGFβ. As we know that this transporter is essential for NK cell metabolism (27), is it possible that TGFβ may also affect the activity of SLC7A5 in NK cells. Overall, it seems clear that TGFβ is directly inhibiting NK cell oxphos and that this is impacting upon their anti-tumor functions. This is likely due to its effects on mitochondrial structure and function such as those described above. Targeting NK cell mitochondrial structure could help restore any TGFβ-induced mitochondrial defects. For example, Buck et al. showed that increasing mitochondrial fusion increases oxphos and anti-tumor functions in T cells (86). This treatment also increased the efficacy of adoptive cell therapy in a model of T cell lymphoma. Similarly, increasing mitochondrial fusion has been shown to increase oxphos in TGFβ and IL10 treated B cells (87). Another way to circumvent the inhibitory effect of TGFβ on oxphos might be to increase the supply of nutrients which can be metabolized by other pathways or to promote mTORC1 activity which preferentially skews metabolism toward glycolysis (e.g., by increasing the availability of amino acids), which seems to be more resistant to TGFβ's negative effects.

Finally, TGFβ has been shown to regulate mitochondrial-endoplasmic reticulum (ER) calcium signaling (88). Under normal conditions, Ca2^+^ is transferred from the ER to the mitochondria where it stimulates ATP production by increasing the activity of electron transport chain dehydrogenase enzymes and increasing proton pumping across the inner membrane. However, treatment of single preglomerular afferent arteriolar smooth muscle cells (PGASMC) with TGFβ reduces the Ca2^+^ release from the ER and impairs the mitochondrial-ER coupling. Indeed, there is evidence supporting the role of calcium in modulating NK cell functions. NK cells from patients with a deficiency in calcium channels have severely impaired degranulation and killing capacity *in vitro* (89). Similarly, Ca2^+^ channel agonists/antagonists have also been shown to inhibit NK cell degranulation and killing capacity (90). Goodridge et al. recently showed that interfering with Ca2^+^ stores reduces degranulation and IFNγ production in human NK cells (91). It seems likely that an interplay between Ca2^+^ signaling, metabolism and function exists in NK cells. The nature of this and the possible role that TGFβ plays in regulating this axis awaits experimental elucidation.

## NK Cell Immunotherapy

**Figure 4:**
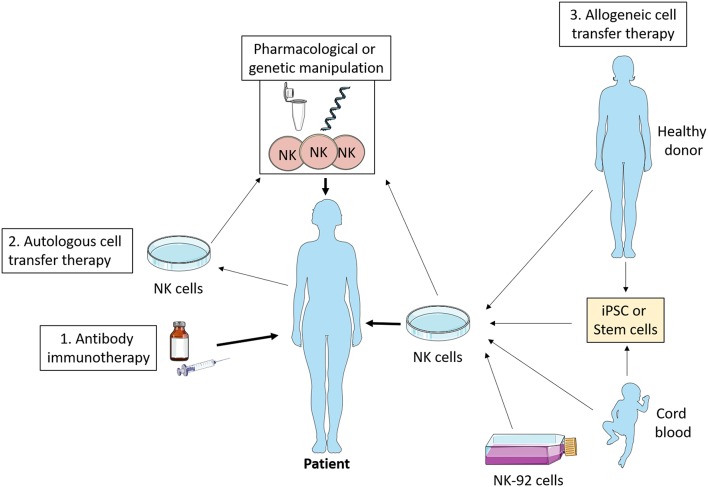
NK cell immunotherapy. Antibody therapy stimulates NK cell ADCC activity *in vivo* which promotes tumor killing. Autologous cell transfer therapy involves treating a patient's own NK cells, while allogeneic cell transfer therapy involves treating cells from a third party—healthy donor, cord blood, stem cells, or NK cell lines. These NK cells can be manipulated *ex vivo* pharmacologically or genetically such that they have enhanced anti-tumor functions and then infused into the patient. iPSC, induced pluripotent stem cells.

NK cells are exploited in a variety of ways therapeutically including as an adoptive cell therapy for cancer. Investigation of NK cell metabolism has the potential to improve this increasingly popular approach. While most studies on adoptive cell therapy have focused on T cells, NK cells offer several advantages (see Figure 4). Unlike T cells, NK cells are generally considered to not be antigen specific, and as such it is more challenging for a tumor to escape the NK cell immune response by antigen mutation alone (92). Importantly, NK cells are less likely to induce graft-vs.-host disease (93), and can positively promote a graft vs. leukemia effect (94, 95). Similarly, NK cells are less likely to promote cytokine release syndrome (96), which has occasionally proven fatal in the context of T cell therapy (97).

As discussed, there is a plethora of ways in which TGFβ is/might be impacting the metabolism of NK cells. This suggests that by blocking the interaction between NK cells and TGFβ we might be able to protect their metabolism and function in TGFβ rich environments, such as those found in cancer. For example, Yvon et al. engrafted a dominant-negative TGFβR2 onto cord blood derived NK cells, leaving them resistant to the inhibitory effects of TGFβ treatment and more efficient at killing glioblastoma tumor cells (98). This was also shown to be effective in the breast cancer setting *in vitro* (99). Similarly, Daher et al. deleted the TGFβR2 in PBMC-derived NK cells using CRISPR-CAS9, rendering them resistant TGFβ and more able to combat a xenograft model of myeloid leukemia (100). We predict that these genetic modifications also enhanced NK cell metabolism, and that this supported the improved anti-tumor immune responses recorded.

While autologous (targeting a patient's own NK cells) NK cell transfer has proven to be safe and well tolerated (101), the most promising anti-tumor responses have been reported with allogeneic (third party) NK cell therapy. This is perhaps because autologous NK cells from cancer patients are often dysfunctional compared to NK cells from healthy individuals. In the case of allogeneic therapy, there have been many promising reports in recent years demonstrating the potential of the treatment. In poor prognosis acute myeloid leukemia (AML) patients, NK cell infusions in combination with high dose chemotherapy and IL2 infusion resulted in *in vivo* expansion of NK cells and complete hematologic remission in 5 out of 19 patients (102). Similar results were more recently shown in elderly high risk AML patients (103), and in high-risk myelodysplastic syndrome (MDS), and again in AML patients (104), where alloreactive NK cells were found in the periphery of all patients and in the bone marrow of some. In neuroblastoma, high doses of NK cell infusions followed by anti-GD2 immunotherapy improved progression free survival, and patient NK cells had increased NKG2A expression (105). In all studies, NK cell infusions were well tolerated, with no reported toxicities. These NK cells were primarily sourced from haploidentical donors i.e., matched for HLA on one chromosome (typically a related donor).

### Future Directions

Indeed, we are in an exciting era of cellular therapy, and basic research such as that described herein will play an essential role in allowing us to harness these new technologies optimally. With the recent advances in genetic engineering, the future possibilities for NK cell immunotherapy are unlimited. Chimeric antigen receptor (CAR)-NK cells are undergoing intense research and are being tested currently in numerous clinical trials for various hematological and solid malignancies (Table 1). So far studies on CAR-NK cells, which have a modified version of surface receptor(s) that allow them to target tumors more efficiently, have been very promising.

**Table 1:** CAR-NK cell clinical trials.

	**Cancer type**
Anti-CD22 CAR NK cells	Refractory B-cell lymphoma
Anti-CD19 CAR NK cells	Refractory B-cell lymphoma
Anti-CD19/CD22 CAR NKCELLS	Refractory B-cell lymphoma
Anti-Mesothelin CAR NK cells	Epithelial ovarian cancer
Anti-PSMA CAR NK cells	Castration-resistant prostate cancer
CAR-NK cells targeting NKG2D ligands	Solid tumors
ROBO1 CAR-NK cells	Solid tumors
BCMA CAR-NK 92 cells	Multiple myeloma
Anti-CD33 CAR-NK cells	Acute myelogenous leukemia, AML, AML with maturation, AML without maturation
Anti-CD19 CAR-NK cells	ALL, CLL, Follicular lymphoma, Mantle cell lymphoma, B-cell prolymphocytic leukemia, Diffuse large cell lymphoma
BiCAR-NK cells (ROBO1 CAR-NK cells)	Pancreatic cancer
BiCAR-NK/T cells (ROBO1 CAR-NK/T cells)	Malignant tumor

*Current clinical trials that involved CAR-NK cells. CD22 and CD19, B cell receptors; Mesothelin, protein expressed in mesothelial cells; PSMA, prostate-specific membrane antigen; ROBO1, roundabout homolog 1; BCMA, B-cell maturation antigen; CD33, myeloid cell antigen; AML, acute myeloid leukemia; CLL, chronic lymphocytic leukemia*.

Li et al. engineered iPSC derived NK cells and T cells to express a CAR construct consisting of the transmembrane domain of NKG2D, the 2B4 co-stimulatory domain (CD244), and the CD3ζ signaling domain (106). These highly active CAR-NK cells and CAR-T cells both exhibited enhanced anti-tumor activity in a murine model of ovarian cancer compared to their non-CAR counterparts. However, the CAR-T cell therapy had significant toxicities which the CAR-NK cell therapy did not e.g., sustained increases in plasma IFNγ, TNFα, and IL2 levels, and pathogenic organ damage in infiltrated organs. At day 70 post treatment, 4/5 mice which received CAR-NK cell therapy were still alive, vs. just 1/5 mice which received CAR-T cell therapy. Similarly, Quintarelli et al. transduced CD19 into *ex vivo* expanded healthy donor NK cells (107), thus allowing them to specifically target transformed B cells. Transfusion of CAR-CD19-NK cells resulted in 100% overall survival vs. 20% for CAR-CD19-T cells in a xenograft model of leukemia in immunodeficient mice. Furthermore, cord blood derived CAR-CD19-NK cells, also transduced with IL15, significantly prolonged survival in a xenograft model of lymphoma (108). There is potential to engineer these CAR-NK cells further to include a modification that boosts metabolism (e.g., increased mTORC1/GAPDH/SLC25A1) or reduces susceptibility to TGFβ (such as those described above).

An important consideration when contemplating new cancer therapies is the economical and physical feasibility of them. Isolation of NK cells from PBMC or stem cells is costly and time-consuming. As such, there is renewed focus on generating “off the shelf” NK cell therapies i.e., a relatively unlimited, generic supply of NK cells that can be banked and then infused into patients when needed. There are several biotechnology companies actively pursuing this therapeutic approach. Interestingly, this idea seems to be moving in the opposite direction to the popular model of “personalized medicine.” NK-92 is a transformed cell line derived from a human NK cell lymphoma patient, and they are known to spontaneously kill cancer cells via direct cytotoxicity and cytokine production. NK-92 cells have also been Food and Drug Administration (FDA) approved for use in clinical trials in 2017 (109). Since then, NK-92 infusions have proven to be well tolerated with only minor toxicities reported in clinical trials for lymphoma and multiple myeloma (110) and advanced renal cell cancer and melanoma patients (111).

NK-92 cells may be genetically engineered such that they have enhanced anti-tumor properties. For example, Yang et al. engrafted a dominant negative TGF?R2 onto NK-92 cells (112) rendering them insensitive to TGFβ. Once transferred to a mouse model of lung cancer, there was reduced tumor proliferation and lung metastasis, while the modified NK-92 cells produced more IFNγ, all of which culminated in increased survival rates. Similarly, Wang et al. genetically engineered the TGF?R2 such that the extracellular and transmembrane region remained intact, yet the intracellular signaling region consisted of intracellular domain of the activating receptor NKD2GD. These NK-92 cells then had enhanced IFNγ production, increased killing capacity *in vivo* and reduced the differentiation of CD4^+^ T cells into Treg cells (113). Beyond TGFβ signaling, NK-92 cells are also amenable to CAR engineering. Oelsner et al. transduced NK-92 cells with CD19 and human CD3ζ. These CAR-NK-92 cells were able to kill tumor cells which their parent cells were resistant to. Furthermore, and in contrast to their non-CAR counterparts, they inhibited progression of an *in vivo* model of lymphoma (114). Indeed, the potential to develop these NK-92 cells as “off the shelf” immunotherapy is immense. Basic research which unveils how cancer impacts NK cells, and how metabolism and cytokines such as TGFβ play into this, will allow us to unlock the full potential of these novel technologies.

### Reimagining NK Cell Mediated Antibody Therapy

NK cell targeted therapy has been in use in the clinic since Rituximab was given FDA approval in 1997 for the treatment of lymphoma. Rituximab, in addition to other antibody therapies such as anti-GD2 and Trastuzumab (for neuroblastoma and Her-2+ breast cancer, respectively), bind to their cognate tumor antigens upon infusion into the patient. NK cells then bind to the Fc region of the antibody via CD16 which initiates ADCC and subsequent killing of the tumor cell. While they have improved survival of these cancers significantly, there is still much room for improvement. For example, combining Herceptin with paclitaxel or with Adriamycin plus cyclophosphamide increased overall survival of HER2^+^ breast cancer patients from 17–41 and 42–56%, respectively (115). While this was a great medical advancement, 41 and 56% overall survival rates are still quite poor. Further, there are many patients who do not respond at all to antibody treatment. As we recently showed that NK cells are metabolically dysfunctional in breast cancer patients (54), we can seek to devise ways to combine current antibody treatments with approaches to increase the metabolism and thus function of the NK cells, thereby improving overall effectiveness of the treatment. One possibility is to harvest patient PBMC, expand the NK cells and increase their metabolism e.g., by inclusion of an inhibitor of TGFβ signaling in cultures. Cells can then be reinfused back into the patient along with antibody therapy. Indeed, TGFβ has been shown to inhibit human NK cell ADCC (30), so we can expect these treated autologous NK cells to also have boosted ADCC activity, which is essential for the antibody therapy to work.

There is also the possibility to accept the fact that patient immune cells are dysfunctional, and to instead use allogeneic adoptive NK cell therapy to mediate the ADCC required for effective antibody mediated therapy. While allogeneic donor NK cells are one possibility, CAR-NK cells could also be an excellent tool in this setting. Jochems et al. engineered NK-92 cells to express the high affinity CD16 receptor (116). These CAR-NK-92 cells then had increased anti-tumor activity and had higher rates of ADCC when used with Herceptin, Cetuximab, and Pertuzumab *in vitro*. Targeting metabolism to improve function and survival of these allogeneic cells in a hostile cancer environment *in vivo* might extend the therapeutic window prior to their eventual rejection. We predict that cell models such as CAR-NK-92 cells could be effective in the treatment of cancers which currently use antibody therapy in their first line of treatment (breast cancer, lymphoma, neuroblastoma etc.). If CAR-NK-92 cells could be developed as “off the shelf” treatment, co-treatment of patients with antibody and CAR-NK-92 cells might become a reality in the years to come.

## Concluding Remarks

Immunometabolism has reinvigorated many aspects of immunology and raised many questions that are now under active investigation. We now know that metabolic dysfunction of immune cells is a key mechanism underlying many human pathologies, including autoimmune disease, infection, obesity and cancer. It is clear that TGFβ treatment inhibits NK cell function and metabolism. In mice, this is mTORC1 dependent, yet in humans it seems that only chronic TGFβ treatment is mTORC1 dependent. Indeed, the interplay between TGFβ and mTORC1 warrants further investigation. It will be exciting to discover what impact TGFβ might have on other important aspects of mitochondrial biology such as ROS production/signaling, mitochondrial dynamics, ER and calcium signaling and much more. Lessons from the literature on other cell types suggest that TGFβ might impact all of these currently unknown aspects of NK metabolism.

The advances that have been made in adoptive cellular therapy and genetic engineering in recent years will no doubt pave the way to new immune therapeutic strategies that are tailored according to cancer type, stage, genotype, immune infiltration and increasingly important, metabolism. Future work must focus on unraveling the molecular pathways that connect these once distant fields of immunology and metabolism, so that we can piece together the best ways to successfully translate immunometabolism to the clinic.

## Author Contributions

The article was written by KS and CG. Figures were mainly prepared by KS.

### Conflict of Interest

The authors declare that the research was conducted in the absence of any commercial or financial relationships that could be construed as a potential conflict of interest.

## Abbreviations

NK: Natural Killer
mTORC1: Mammalian target of rapamycin complex 1
ADCC: Antibody dependent cellular cytotoxicity
2DG: 2-deoxyglucose
TCA: Tricarboxylic acid
DC: Dendritic cell
GAPDH: Glyceraldehyde 3-phosphate dehydrogenase
ROS: Reactive oxygen species
PGC-1α: Proliferator-activated receptor gamma coactivator 1-alpha
SREBP: Sterol regulatory element-binding protein
IRE1: Inositol-requiring enzyme 1
TGFβR2: TGFβ receptor 2
TGFβR1: TGFβ receptor 1
FBP1: Fructose-1,6-bisphosphatase
AML: Acute myeloid leukaemia
MDS: Myelodysplastic syndrome
iPSC: Induced pluripotent stem cells
SOD: Superoxide dismutase
NAC: N-acetylcysteine
NOX4: NADPH oxidase 4
UCP2: Uncoupling protein 2
ER: Endoplasmic reticulum
PGASMC: Preglomerular afferent arteriolar smooth muscle cells
CAR: Chimeric antigen receptor
FDA: Food and Drug Administration
ATP: Adenosine tri-phosphate.
